# Image-domain deep learning denoising for low-dose chest CT on a single 128-slice CT platform: a retrospective image-quality assessment

**DOI:** 10.3389/fmed.2026.1888006

**Published:** 2026-07-14

**Authors:** Kaiqing Yao, Xue Jiang, Liang Lv, Yang Li, Guangpeng Zhang, Zhiyuan Zhang, Zhiwei Zhang, Xinyou Li, Fajin Lv

**Affiliations:** 1 Department of Radiology, The First Affiliated Hospital of Chongqing Medical University, Chongqing, China; 2 Department of Radiology, Chongqing Academy of Medical Sciences, Chongqing General Hospital, Chongqing University, Chongqing, China; 3 Department of Physical Algorithm, Sinovision Technologies (Beijing) Co., Ltd., Beijing, China; 4 Department of Safety Assessment, Chongqing Changming Safety Technology Consulting Co., Ltd., Chongqing, China

**Keywords:** image quality, iterative reconstruction, low-dose chest CT, radiation dose optimisation, vendor-independent image-domain deep learning denoising

## Abstract

**Background:**

Image-domain deep learning denoising may provide a practical post-processing approach for improving low-dose chest CT image quality on scanners without native deep learning reconstruction. This retrospective study evaluated a vendor-independent image-domain denoising algorithm applied to low-dose chest CT on a single 128-slice CT platform and descriptively compared the resulting image-quality metrics with those obtained using a standard-dose iterative reconstruction protocol.

**Methods:**

This retrospective study included 198 patients who underwent unenhanced chest CT and were assigned to a low-dose CT group (LDCT, *n* = 99) or a standard-dose CT group (SDCT, *n* = 99) according to the clinical acquisition protocol. All images were reconstructed using sinogram-affirmed iterative reconstruction (SAFIRE). LDCT images were additionally processed with a vendor-independent image-domain deep learning denoising algorithm (AiR Denoising), generating low-dose AiR-denoised images (LD-AiR). Objective image quality was assessed using attenuation, image noise, signal-to-noise ratio, and contrast-to-noise ratio. Subjective image quality of the lung parenchyma and mediastinal soft tissue was independently evaluated by readers using a 5-point scale.

**Results:**

Compared with low-dose SAFIRE (LD-SAFIRE), LD-AiR significantly reduced image noise and increased signal-to-noise ratio and contrast-to-noise ratio across all evaluated regions (all *p* < 0.05). Subjective image quality scores for both lung parenchyma and mediastinal soft tissue were also significantly higher with LD-AiR than with LD-SAFIRE (all *p* < 0.05). In the descriptive between-group comparison, the LDCT protocol was associated with an approximately 76% lower effective dose than the SDCT protocol. Most objective and subjective image-quality metrics of LD-AiR did not differ significantly from those of SD-SAFIRE; however, this comparison was based on different patient groups and should not be interpreted as evidence of equivalence or non-inferiority.

**Conclusion:**

On the evaluated CT platform, vendor-independent image-domain deep learning denoising improved objective and subjective image quality of low-dose chest CT compared with LD-SAFIRE. These findings suggest that image-domain denoising may have potential utility for image-quality improvement on CT systems without native deep learning reconstruction. Further prospective, within-subject, multicentre studies incorporating lesion-detectability and diagnostic-performance assessment are needed to clarify its clinical applicability.

## 1 Introduction

Low-dose chest computed tomography (LDCT) has become an important tool in thoracic imaging and is used in a variety of clinical settings, including lung cancer screening and the follow-up evaluation of pulmonary lesions (1, 2). However, dose reduction generally increases image noise and susceptibility to artefacts, which may impair lesion conspicuity and obscure fine anatomical details (3). Iterative reconstruction (IR) has therefore been widely adopted in LDCT to mitigate these limitations; nevertheless, achieving an optimal balance between noise reduction and preservation of natural image texture remains challenging (3, 4). At higher reconstruction strengths, IR may produce overly smooth or blotchy images with altered noise characteristics, potentially reducing diagnostic confidence (4).

Deep learning-based strategies, including deep learning reconstruction and image-domain post-processing denoising approaches, have been increasingly investigated for image-quality improvement in LDCT (5). Recent reviews have highlighted the increasing significance of these deep learning-based methods in CT imaging, particularly in minimizing image noise while maintaining natural image texture at reduced radiation dose levels (6, 7). In alignment with these observations, prior research has indicated that such approaches may effectively reduce image noise while preserving texture and structural detail, in comparison to conventional IR techniques (8–10). However, the majority of existing clinical evidence has predominantly centered on vendor-specific deep learning reconstruction (DLR) methods integrated into select CT platforms, thereby constraining the applicability of these approaches to numerous installed scanners that lack native DLR capabilities.

Thus, vendor-independent image-domain deep learning denoising may serve as a viable post-processing option for current CT systems. Within the broader context of deep learning-based image-quality improvement in CT (5), recent studies have shown that these algorithms can be implemented in the image domain without necessitating scanner-integrated DLR (11, 12). More recent investigations have begun to assess deep learning–based denoising approaches in clinical CT imaging, reporting improvements in image quality metrics while maintaining diagnostic performance in certain scenarios (13). This approach may be particularly pertinent for routine low-dose chest CT conducted on scanners that do not support native DLR. Nonetheless, clinical evidence regarding vendor-independent post-processing remains limited, and the comparison of its image quality metrics with those derived from standard-dose IR has not been thoroughly examined within routine chest CT practice (11, 12).

Accordingly, this retrospective study aimed to evaluate whether vendor-independent image-domain deep learning denoising could improve objective and subjective image quality of routine low-dose chest CT on a single CT scanner without native DLR capability and to descriptively investigate how its image quality metrics compare with those obtained using standard-dose IR. The study focused on image-quality endpoints and was not designed to establish equivalence, non-inferiority, lesion detectability, or diagnostic performance.

## 2 Materials and methods

### 2.1 Study population

This retrospective study involved 198 eligible patients who underwent unenhanced chest CT at the First Affiliated Hospital of Chongqing Medical University from January 2020 to January 2025. Patients were classified into two groups: the low-dose CT group (LDCT, *n* = 99) and the standard-dose CT group (SDCT, *n* = 99), according to the clinical acquisition protocol used in routine practice. Group allocation was based on the clinically selected CT protocol and was not randomized. Therefore, the LDCT and SDCT groups represented two real-world protocol cohorts rather than experimentally matched groups. Individuals with missing thin-section images or non-diagnostic image quality due to motion or respiratory artifacts were excluded from the study.

Because body mass index was not available for all patients, maximum anteroposterior thoracic diameter, maximum transverse thoracic diameter, and scan length were used as surrogate indicators of body habitus and scan coverage. These variables were compared between the LDCT and SDCT groups to assess baseline comparability.

Clinical indications for chest CT examinations were retrospectively extracted from the picture archiving and communication system/radiology information system (PACS/RIS) examination request forms and categorized according to the predominant clinical reason for the examination. The categories included pulmonary nodule screening or follow-up, infection or inflammation assessment, chest symptom evaluation, tumor-related evaluation or follow-up, preoperative or routine inpatient assessment, and other or unclear indications. When more than one indication was listed, the predominant clinical reason for the examination was used for classification.

Sample size estimation was performed using PASS 15, focusing on a two-sample comparison of means with a two-sided significance level of 0.05 and a statistical power of 90%. The estimation was based on preliminary data from 51 patients in each group, with lung image noise selected as the reference image-quality parameter. The minimum required sample size was determined to be 81 patients per group.

**Figure 1:**
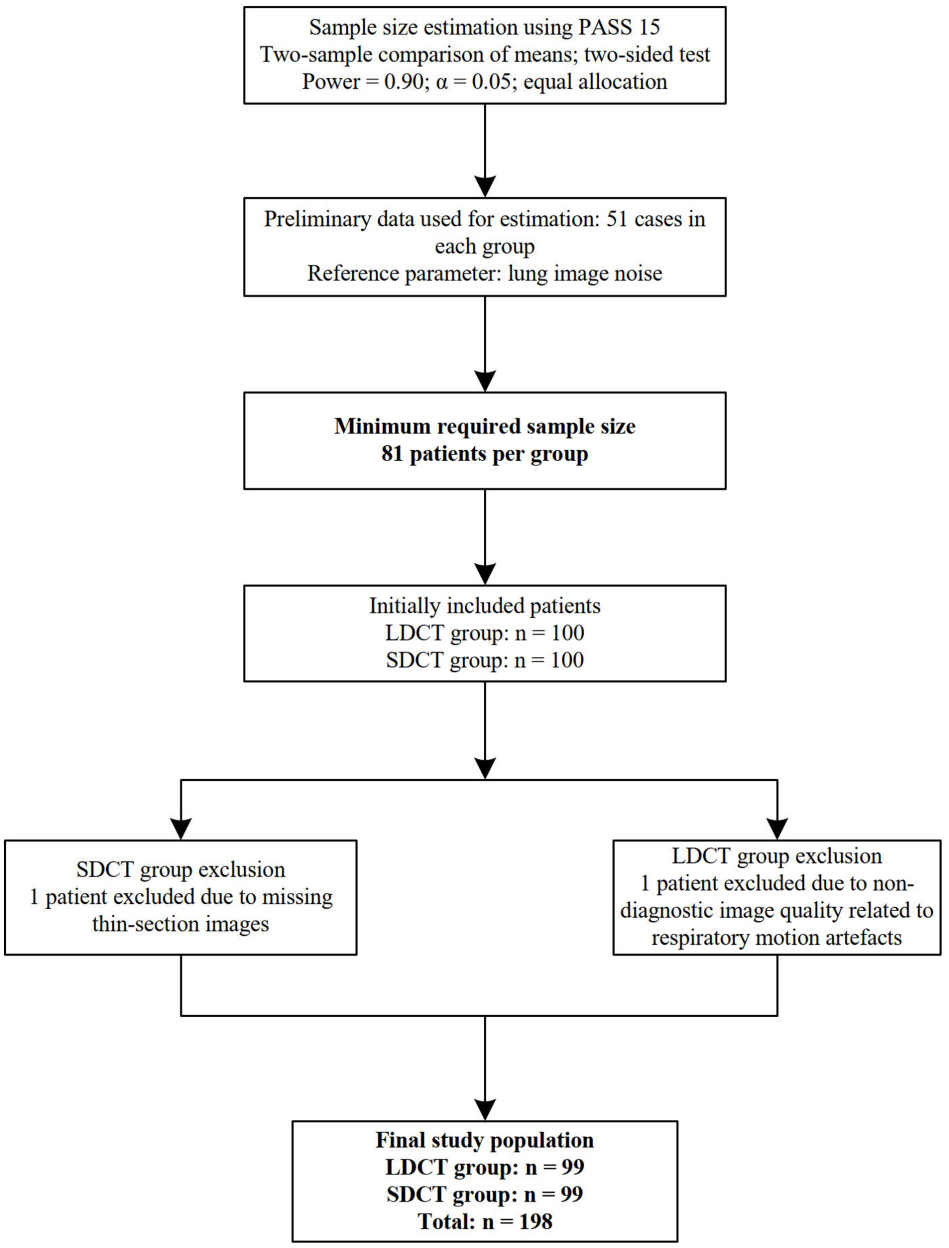
Flowchart of sample size estimation, patient selection, and final study population.

Therefore, 100 patients were initially included in each group. However, one SDCT case was excluded due to missing thin-section images, and one LDCT case was excluded because of non-diagnostic image quality resulting from respiratory motion artefacts. Thus, the final analysis comprised 99 patients in each group (Figure 1).

The Institutional Review Board of the First Affiliated Hospital of Chongqing Medical University approved this retrospective study (Approval No. 2025–606-01; approved on 2 September 2025). Ethics approval was secured prior to the extraction and analysis of retrospective data. The requirement for written informed consent was waived by the Institutional Review Board due to the study’s retrospective nature.

### 2.2 CT scanning and image reconstruction

Chest CT examinations were performed using a 128-slice CT system (SOMATOM Perspective; Siemens Healthineers, Forchheim, Germany) equipped with CARE Dose 4D automatic tube current modulation. For the low-dose protocol, the tube voltage was 110 kVp, the quality reference tube current–time product was 20 mAs, and the pitch was 1.3. For the standard-dose protocol, the tube voltage was 130 kVp, the quality reference tube current–time product was 80 mAs, and the pitch was 1.1. The CARE Dose 4D modulation strength for the chest protocol was Average. The quality reference mAs represented the protocol-defined reference value used by the automatic exposure control system, whereas the actual tube current–time product varied according to patient attenuation and automatic tube current modulation during scanning.

Patients were positioned supine with both arms raised above the head. An anteroposterior localizer radiograph was obtained before helical acquisition. Patient positioning and centering were performed according to the routine institutional chest CT workflow by trained radiographers. In the lateral direction, centering was performed along the mid-axillary line, and the craniocaudal scan range was planned from the lower neck or suprasternal region to cover the entire chest. No quantitative off-centering measurement was performed retrospectively. The reconstruction field of view was adjusted by the radiographers according to patient body habitus to include the entire thorax rather than being fixed across all patients.

All other acquisition parameters were consistent across both protocols, including a detector collimation of 64 mm × 0.6 mm, a rotation time of 0.6 s, and a reconstruction matrix of 512 × 512. Images were reconstructed using sinogram-affirmed iterative reconstruction (SAFIRE; strength level 3), with an I50s kernel, a section thickness of 1.0 mm, and an increment of 1.0 mm. SAFIRE strength level 3 and the I50s kernel were used because they represented the routine institutional chest CT reconstruction setting on this CT platform. Because the LDCT and SDCT protocols differed in acquisition settings, the analysis comparing low-dose AiR-denoised images (LD-AiR) with standard-dose SAFIRE (SD-SAFIRE) analysis was interpreted as a descriptive protocol-level comparison.

For the thin-section images obtained from the LDCT group, additional post-processing was applied using a vendor-independent, image-domain deep learning denoising algorithm (AiR Denoising, version 1.0; Sinovision Technologies, Beijing, China). These processed images were designated as LD-AiR. The algorithm used reconstructed Digital Imaging and Communications in Medicine (DICOM) images as input and generated denoised image-domain CT images as output, without requiring access to raw projection data or sinogram data. A fixed vendor-provided denoising setting was applied to all LDCT images, and no case-specific manual adjustment was performed. The algorithm aimed to reduce image noise while preserving anatomical detail and maintaining a noise texture similar to that of filtered back projection. Detailed network architecture and training-data composition were not fully disclosed to the investigators, which may limit technical reproducibility and external validation.

### 2.3 Radiation dose assessment

For each CT examination, the tube voltage, tube current, volume CT dose index (CTDIvol), and dose–length product (DLP) were obtained from the scanner-generated dose report. The effective dose (ED) was estimated from DLP using a chest-specific conversion coefficient of 0.014 mSv·mGy − 1·cm − 1, as applied in previous chest CT studies (10, 14, 15). The ED was calculated as follows: ED = DLP × 0.014.

Size-specific dose estimate (SSDE) was additionally calculated as a supplementary dose metric using the effective-diameter method described in American Association of Physicists in Medicine (AAPM) Report 204 (16). The anteroposterior and transverse thoracic diameters measured at the level of the twelfth thoracic vertebra were used to estimate patient size. For SSDE estimation, the mean anteroposterior and transverse diameters from the two independent readers were used. The effective diameter was calculated as the square root of the product of the mean anteroposterior and transverse diameters. SSDE was calculated by multiplying scanner-reported CTDIvol by the corresponding size-specific conversion factor for the 32-cm body CTDI phantom.

### 2.4 Image quality assessment

All image datasets were transferred to a dedicated workstation (United Imaging, Shanghai, China) for analysis. This study focused on objective and subjective image-quality assessment and did not include lesion-detectability or diagnostic-performance evaluation. Three independent readers conducted the image quality assessments. Reader A, a dual-qualified radiological technologist and radiologist with 13 years of experience, participated in both subjective and objective assessments. Reader B, a radiological technologist with 16 years of experience, performed the objective assessment only. Reader C, a radiologist with 7 years of experience, performed the subjective assessment only.

To minimize inter-reader variability arising from differences in professional backgrounds, all readers underwent standardized training and calibration prior to the study. Image review and scoring were executed in a randomized order. All readers were blinded to the CT dose protocol and reconstruction/post-processing labels, and the DICOM metadata were removed before image review. However, because LD-AiR images may have distinctive post-processing characteristics, complete blinding to reconstruction type could not be fully guaranteed.

#### 2.4.1 Thoracic diameters and scan length

Readers A and B measured the maximum transverse thoracic diameter, the maximum anteroposterior thoracic diameter, and the scan length using low-dose SAFIRE (LD-SAFIRE) and SD-SAFIRE images. Thoracic diameters were assessed on axial images at the level of the twelfth thoracic vertebra, while the scan length was measured on coronal images. These measurements were used as surrogate indicators of body habitus and scan coverage because body mass index was not available for all patients.

#### 2.4.2 Objective image quality assessment

Two readers (Readers A and B) conducted an independent objective image quality assessment on three image sets: LD-SAFIRE, SD-SAFIRE, and LD-AiR. For each region of interest (ROI), the mean attenuation value, expressed in Hounsfield units (HU), along with its standard deviation (SD), was documented. The SD of the attenuation value was interpreted as image noise. Signal-to-noise ratio (SNR) and contrast-to-noise ratio (CNR) were derived from these measurements (8, 17). In this study, CNR was calculated using a previously published noise-weighted formula applied in chest CT deep learning reconstruction studies; therefore, CNR values were interpreted only for relative comparisons within this study.

ROIs of approximately 100 mm^2^ were positioned on a single axial image at specific anatomical locations, which included normal lung parenchyma in the right lung adjacent to an interlobar fissure, the descending thoracic aorta with the ROI centred within the lumen while excluding the vessel wall, the right infraspinatus muscle, segment VII of the liver, and the body of the twelfth thoracic vertebra. Care was taken to avoid including vessels, bile ducts, focal lesions, and visible artifacts. Background noise was measured in air located 3–5 cm anterior to the sternal margin (17).

To enhance measurement consistency, ROI placement was standardized using fixed anatomical landmarks for each structure. In the paired comparison between LD-SAFIRE and LD-AiR within the LDCT group, the ROI drawn on one image set was transferred to the corresponding image on the same axial level and at identical coordinates in the other image set, ensuring spatially matched measurements between the two reconstructions. The two readers independently performed ROI placement rather than relying on a shared ROI template. For the SD-SAFIRE images, ROIs were independently placed at the corresponding predefined anatomical locations using the same placement rules.

Because the assessment was based on single-slice measurements, each ROI represented one predefined axial level rather than an average across multiple slices. This approach was used to maintain consistency with previous image-quality studies, although it may not fully capture regional heterogeneity across the entire scanned volume.

The SNR was calculated using Equation 1:


$$\mathrm{SNR}=\frac{{\mathrm{HU}}_{\mathrm{ROI}}}{{\mathrm{SD}}_{\mathrm{ROI}}}$$ (1)

Because attenuation values in the lung are negative on the Hounsfield unit scale, the absolute value of HU_ROI_ was used to express signal magnitude.

The CNR was calculated using Equation 2:


$$\mathrm{CNR}=\frac{2{\left({\mathrm{HU}}_{\text{Target}}-{\mathrm{HU}}_{\text{Background}\;\;\mathrm{air}}\right)}^{2}}{{\mathrm{SD}}_{\text{Target}}^{2}+{\mathrm{SD}}_{\text{Background}\;\;\mathrm{air}}^{2}}$$ (2)

According to previously published chest CT deep learning reconstruction studies (8, 17). This formula was retained to maintain methodological consistency with prior studies. Because it differs from the conventional CNR definition and includes a squared attenuation difference relative to background air, the resulting absolute values are not directly comparable with conventional CNR values. Therefore, only relative differences among LD-SAFIRE, LD-AiR, and SD-SAFIRE were interpreted.

Due to its formulation, which includes a squared attenuation difference in relation to background air, this metric yields absolute values that exceed those typically derived from standard CNR definitions. Consequently, the focus was placed solely on relative comparisons between the reconstruction methods.

#### 2.4.3 Subjective image quality assessment

Readers A and C independently evaluated the subjective image quality of the lung parenchyma and mediastinal soft tissues across all image sets using a 5-point Likert scale. The images were assessed in a randomized order, with the readers blinded to the scan parameters and reconstruction methods. The subjective assessment focused on overall image quality and anatomical structure visibility rather than lesion detection or diagnostic accuracy. However, due to the distinctive post-processing characteristics of LD-AiR images, complete blinding to the reconstruction type could not be fully guaranteed.

For assessing lung parenchymal image quality, the scoring criteria were as follows: 1, pulmonary markings poorly visualised (unacceptable); 2, pulmonary markings insufficiently clear (poor); 3, pulmonary markings moderately clear (acceptable); 4, pulmonary markings clearly visualised (good); and 5, pulmonary markings very clearly visualised (optimal). Similarly, for mediastinal soft-tissue image quality, the scoring was defined as: 1, mediastinal structures poorly visualised (unacceptable); 2, mediastinal structures insufficiently clear (poor); 3, mediastinal structures moderately clear (acceptable); 4, mediastinal structures clearly visualised (good); and 5, mediastinal structures very clearly visualised (optimal) (17).

### 2.5 Statistical analysis

Statistical analyses were performed using SPSS software (version 32.0; IBM Corp., Armonk, NY, USA). Normality was evaluated with the Shapiro–Wilk test. Continuous variables that followed a normal distribution are presented as the mean ± standard deviation (SD), whereas non-normally distributed variables are presented as the median and interquartile range (IQR). Categorical variables are reported as frequencies and percentages.

Two predefined image-based comparisons were performed: LD-SAFIRE versus LD-AiR, and LD-AiR versus SD-SAFIRE. The paired LDCT comparison was defined as the primary image-quality analysis and included objective image noise, SNR, CNR, and subjective image-quality scores as primary endpoints. Attenuation measurements and analyses of individual anatomical regions were considered supportive image-quality assessments. The LD-AiR versus SD-SAFIRE comparison was exploratory and descriptive because the image sets were obtained from different patient groups and different acquisition protocols. The comparison between LD-SAFIRE and LD-AiR employed either the paired t-test or the Wilcoxon signed-rank test, as appropriate. For paired comparisons, paired differences were used to support interpretation of the direction and magnitude of change between LD-SAFIRE and LD-AiR. Where appropriate, nonparametric 95% confidence intervals (CIs) for median paired differences were calculated using order statistics to describe the precision of the observed changes.

The comparison between LD-AiR and SD-SAFIRE was performed between different patient groups and used either the independent-samples *t*-test or the Mann–Whitney U test, as appropriate. This analysis was considered a descriptive between-group protocol-level comparison because the LDCT and SDCT groups consisted of different patients and were acquired using different clinical protocols. No formal equivalence or non-inferiority testing was performed; therefore, non-significant *p* values in the LD-AiR versus SD-SAFIRE comparison were not interpreted as evidence of equivalence or non-inferiority.

The chi-square test was used to assess differences in sex between the LDCT and SDCT groups. The distribution of clinical indications between the LDCT and SDCT groups was compared using the Fisher–Freeman–Halton exact test because some categories contained small expected cell counts. Cramér’s V was reported as a measure of effect size for the contingency-table analysis. Because multiple image-quality metrics and anatomical regions were evaluated, *p* values were interpreted descriptively in the context of multiple image-quality comparisons. All statistical tests were two-sided, with a *p* value < 0.05 indicating statistical significance.

Inter-reader reliability for continuous objective measurements was assessed using the intraclass correlation coefficient (ICC). A two-way mixed-effects consistency model was applied, and single-measure ICC values were reported. ICC values were interpreted as poor (<0.50), moderate (0.50–0.75), good (0.75–0.90), or excellent (>0.90) (18). Because the subjective image-quality scores were ordinal, inter-reader agreement was assessed using linearly weighted kappa (*κ*) statistics. Weighted κ values were interpreted using commonly accepted thresholds as slight (≤0.20), fair (0.21–0.40), moderate (0.41–0.60), substantial (0.61–0.80), or almost perfect (0.81–1.00) (2, 9, 19).

## 3 Results

### 3.1 General characteristics of the study population

No statistically significant differences were observed between the LDCT and SDCT groups in sex, age, maximum anteroposterior thoracic diameter, maximum transverse thoracic diameter, or scan length (all *p* > 0.05). Because body mass index was not available for all patients, thoracic diameters and scan length were used as surrogate indicators of body habitus and scan coverage. These parameters were also comparable between the two protocol cohorts. Inter-reader agreement for measurements of thoracic diameter and scan length was excellent, with ICC values ranging from 0.995 to 1.000. Detailed results are presented in Table 1 and Supplementary Table S1.

**Table 1:** Baseline characteristics of the LDCT and SDCT groups.

Variable	LDCT group (*n* = 99)	SDCT group (*n* = 99)	Test statistic	*p*-value
Age, years	49.00 (23.00)	47.00 (23.00)	−0.45	0.653
Sex, n (%)			0.19	0.662
Male	40 (40.4)	37 (37.4)		
Female	59 (59.6)	62 (62.6)		

Age is presented as median (interquartile range), and sex is presented as number (%). The test statistic denotes the Z or χ^2^ value, as appropriate. All *p*-values were two-sided. Additional body-size-related and scan-coverage parameters are provided in Supplementary Table S1.

Infection or inflammation assessment was the most common clinical indication in both groups. The distribution of clinical indications did not differ significantly between the LDCT and SDCT groups (Fisher–Freeman–Halton exact test, *p* = 0.444; Cramér’s V = 0.159). Detailed results are provided in Supplementary Table S2.

### 3.2 Comparison of radiation dose parameters

The tube current, CTDIvol, DLP, and ED were all significantly lower in the LDCT group than in the SDCT group (all *p* < 0.001; Table 2). The LDCT protocol was associated with an approximately 76% lower ED than the SDCT protocol. This difference reflected the predefined routine acquisition protocols rather than an isolated effect of image-domain denoising. In the supplementary SSDE analysis, effective diameter was comparable between the two groups (*p* = 0.429), whereas SSDE remained significantly lower in the LDCT group than in the SDCT group (*p* < 0.001). Detailed results are provided in Supplementary Table S3.

**Table 2:** Comparison of radiation dose parameters between the LDCT and SDCT groups.

Group	n	Tube voltage, kV	Tube current, mAs	CTDIvol, mGy	DLP, mGy·cm	ED, mSv
LDCT	99	110	20.00 (5.00)	1.43 (0.41)	56.48 ± 11.08	0.79 ± 0.16
SDCT	99	130	55.19 ± 11.77	6.14 ± 1.29	235.25 ± 53.72	3.29 ± 0.75
Test statistic			−12.17	−12.16	−32.43	−32.45
*p*-value			<0.001	<0.001	<0.001	<0.001

Data are presented as mean ± standard deviation or median (interquartile range), as appropriate. The test statistic denotes the *t* or *Z* value, as appropriate. Tube voltage was protocol-defined and was therefore not included in the statistical comparison. CTDIvol indicates volume CT dose index; DLP, dose–length product; and ED, effective dose. Dose differences should be interpreted as protocol-level differences between the LDCT and SDCT acquisition protocols rather than as an isolated effect of image-domain denoising.

### 3.3 Comparison of objective attenuation measurements

Inter-reader reliability for mean attenuation measurements in the lung, liver, and vertebral body was found to be good to excellent (ICC, 0.753–0.958). In contrast, the agreement for measurements taken in the aorta, muscle, and background air exhibited more variability, with ICC values ranging from poor to good (ICC, 0.318–0.773).

In the LDCT group, significant differences in attenuation values were observed between LD-SAFIRE and LD-AiR across the lung, aorta, muscle, liver, vertebral body, and background air (all *p* < 0.05). However, these statistically significant differences should be interpreted in the context of their small numerical magnitude. Supplementary Table S4 demonstrated that the paired attenuation differences were small in magnitude for all tissue ROIs, with median ΔHU values ranging from −1.6 to −0.3 HU and mean absolute differences from 0.8 to 1.7 HU across readers. In addition, 98–100% of paired measurements for these tissue ROIs fell within ±5 HU, suggesting limited practical relevance for routine tissue attenuation assessment despite statistical significance. In contrast, background air showed a relatively larger shift (median ΔHU, 5.0–5.2 HU), with only 47.5–50.5% of measurements maintaining ±5 HU.

When comparing LD-AiR with SD-SAFIRE, significant differences in attenuation were noted in muscle, vertebral body, and background air for both readers (all *p* < 0.05). Reader A also identified a significant difference in the aorta (*p* = 0.014); however, no significant differences were observed in the lung or liver for either reader, nor in the aorta for Reader B (*p* = 0.091). Because LD-AiR and SD-SAFIRE were derived from different patient groups and different acquisition protocols, these between-group findings were interpreted descriptively rather than as evidence for equivalence or non-inferiority. Detailed results are presented in Supplementary Table S5 and Supplementary Figure S1.

### 3.4 Comparison of objective noise measurements

Inter-reader reliability for image noise measurements in the lung ranged from poor to moderate (ICC, 0.487–0.734). Agreement for the aorta was good (ICC, 0.775–0.887), while agreement for the liver ranged from moderate to good (ICC, 0.710–0.870). Agreement for muscle, vertebral body, and background air was more variable, ranging from poor to good (ICC, 0.344–0.757).

Within the LDCT group, LD-AiR demonstrated significantly lower SD values values compared to LD-SAFIRE images in the lung, aorta, muscle, liver, vertebral body, and background air for both readers (all *p* < 0.05), indicating lower measured image noise.

**Figure 2:**
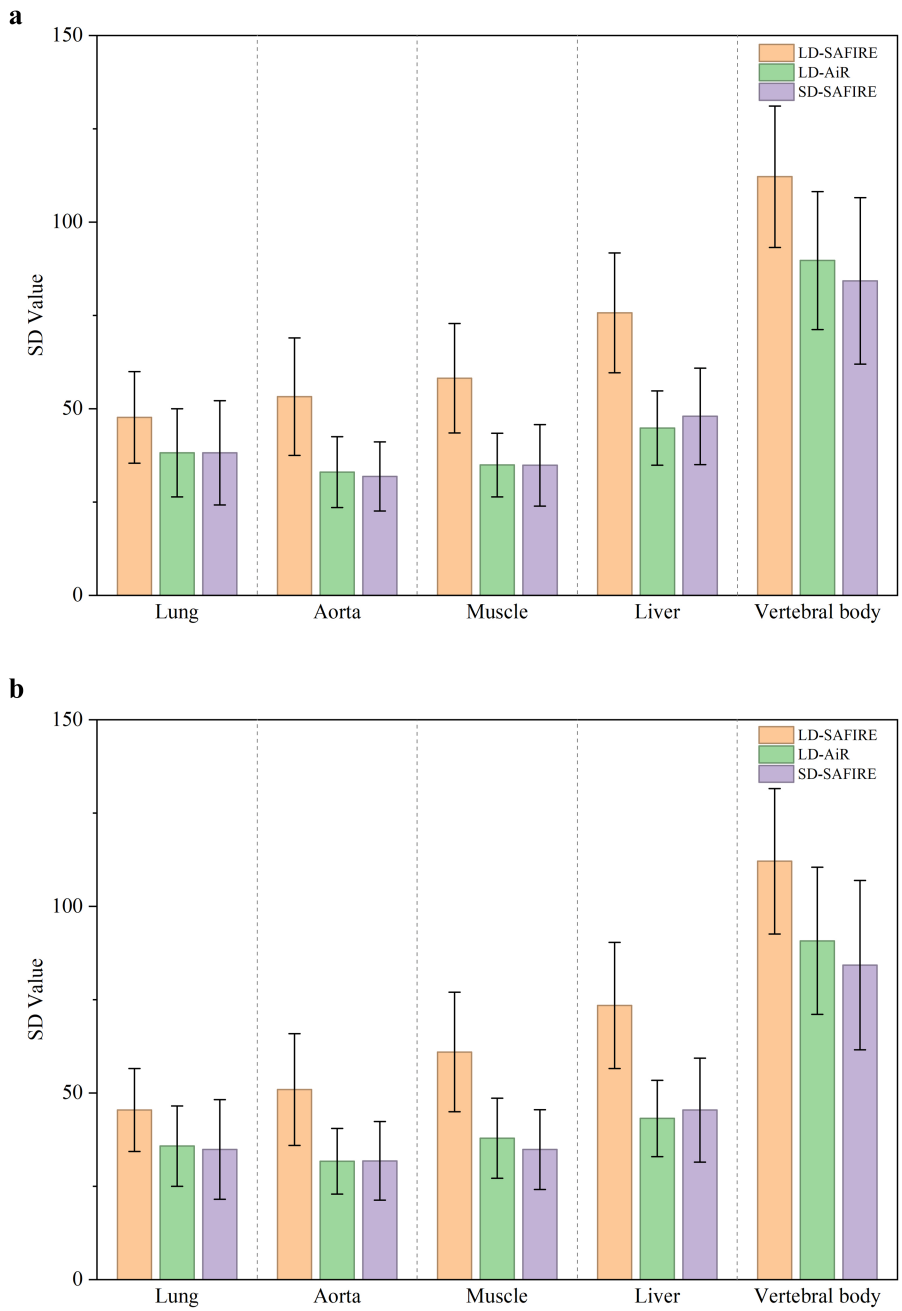
Image noise across reconstruction methods. Bar plots show image noise measured by Reader A **(a)** and Reader B **(b)** across anatomical regions in the LD-SAFIRE, LD-AiR, and SD-SAFIRE image sets. Error bars indicate standard deviation or interquartile range, as appropriate.

In the between-group comparison between LD-AiR and SD-SAFIRE, no statistically significant differences were observed for most image-noise measurements across the assessed structures. The only exceptions were muscle image noise measured by Reader B (*p* = 0.049) and vertebral body image noise measured by Reader B (*p* = 0.017). Because LD-AiR and SD-SAFIRE were obtained from different patient groups and different acquisition protocols, these between-group results were interpreted descriptively. Detailed results are presented in Table 3 and Figure 2.

**Table 3:** Comparison of image noise among LD-SAFIRE, LD-AiR, and SD-SAFIRE images.

Image noise, HU	LD-SAFIRE	LD-AiR	SD-SAFIRE	Δpaired	95% CI for median Δpaired	*p*1	*p*2
Lung							
ICC	0.531	0.487	0.734				
Reader A	45.50 (11.50)	36.90 (13.20)	38.17 ± 13.94	−9.20 (5.45)	−9.70 to −8.30	<0.001	0.992
Reader B	44.10 (13.10)	34.40 (14.10)	34.85 ± 13.37	−9.40 (6.40)	−10.00 to −8.00	<0.001	0.611
Aorta							
ICC	0.887	0.824	0.775				
Reader A	53.19 ± 15.74	33.00 ± 9.50	31.85 ± 9.28	−20.50 (9.15)	−21.40 to −18.80	<0.001	0.391
Reader B	50.9 ± 14.98	31.20 (13.60)	29.60 (15.10)	−18.60 (9.70)	−20.60 to −17.40	<0.001	0.761
Muscle							
ICC	0.534	0.518	0.757				
Reader A	58.17 ± 14.65	34.92 ± 8.52	33.60 (15.30)	−23.10 (10.10)	−25.20 to −21.30	<0.001	0.607
Reader B	60.96 ± 16.01	37.86 ± 10.72	34.84 ± 10.71	−22.40 (8.15)	−24.20 to −20.90	<0.001	0.049
Liver							
ICC	0.781	0.710	0.870				
Reader A	72.50 (19.10)	42.70 (12.70)	47.95 ± 12.94	−29.60 (9.35)	−32.20 to −28.00	<0.001	0.112
Reader B	70.50 (21.90)	42.00 (10.20)	44.50 (23.00)	−28.30 (10.00)	−31.00 to −27.10	<0.001	0.367
Vertebral body							
ICC	0.600	0.539	0.653				
Reader A	112.14 ± 18.96	89.68 ± 18.49	84.22 ± 22.29	−21.50 (13.55)	−23.40 to −19.00	<0.001	0.062
Reader B	112.10 ± 19.48	87.10 (25.80)	84.26 ± 22.71	−20.70 (12.85)	−24.20 to −18.10	<0.001	0.017
Background air							
ICC	0.344	0.346	0.472				
Reader A	31.90 (45.20)	18.60 (52.20)	20.70 (39.70)	−6.80 (8.10)	−8.10 to −4.30	<0.001	0.516
Reader B	29.90 (31.40)	18.20 (40.00)	22.30 (44.40)	−7.40 (7.90)	−9.40 to −6.40	<0.001	0.098

Data are presented as mean ± standard deviation or median (interquartile range), as appropriate. ICC indicates inter-reader reliability. Δpaired was calculated as LD-AiR minus LD-SAFIRE and is presented as median (interquartile range), reflecting within-patient paired differences in the LDCT group. The 95% CI refers to the nonparametric 95% confidence interval for the median paired difference. *p*1 denotes the paired comparison between LD-SAFIRE and LD-AiR, performed using the paired t-test or Wilcoxon signed-rank test, as appropriate. *p*2 denotes the between-group comparison between LD-AiR and SD-SAFIRE, performed using the independent-samples *t*-test or Mann–Whitney U test, as appropriate. All *p*-values were two-sided.

### 3.5 Comparison of SNR and CNR

Within the LDCT group, LD-AiR demonstrated significantly higher SNR and CNR than LD-SAFIRE in the lung, aorta, muscle, liver, and vertebral body (all *p* < 0.05). For lung measurements, SNR was expressed as absolute SNR; thus, the higher values observed for LD-AiR indicate greater absolute signal relative to image noise. Similar increases were noted across all assessed structures for both readers.

In the between-group comparison between LD-AiR and SD-SAFIRE, no statistically significant differences were observed for most SNR measurements across the evaluated structures. The only significant difference was liver SNR measured by Reader A (*p* = 0.035). For CNR, no statistically significant differences were observed between LD-AiR and SD-SAFIRE for any evaluated structure (all *p* > 0.05). These between-group results were interpreted descriptively because LD-AiR and SD-SAFIRE were derived from different patient groups and different acquisition protocols. Detailed results are presented in Tables 4, 5, with corresponding graphical summaries shown in Supplementary Figures S2, S3.

**Table 4:** Comparison of SNR among LD-SAFIRE, LD-AiR, and SD-SAFIRE images.

SNR	LD-SAFIRE	LD-AiR	SD-SAFIRE	Δpaired	95% CI for median Δpaired	*p*1	*p*2
Lung							
Reader A	19.44 (5.34)	23.79 (9.93)	23.22 (12.85)	4.71 (4.85)	4.12 to 5.25	<0.001	0.938
Reader B	20.72 ± 5.34	26.03 (10.78)	25.75 (19.50)	5.16 (5.76)	4.49 to 6.19	<0.001	0.617
Aorta							
Reader A	0.86 ± 0.33	1.41 ± 0.51	1.36 ± 0.53	0.54 (0.27)	0.47 to 0.59	<0.001	0.516
Reader B	0.81 (0.52)	1.37 (0.71)	1.42 ± 0.52	0.55 (0.25)	0.50 to 0.60	<0.001	0.484
Muscle							
Reader A	0.94 (0.47)	1.71 ± 0.57	1.46 (0.83)	0.69 (0.33)	0.60 to 0.73	<0.001	0.110
Reader B	0.94 ± 0.31	1.57 ± 0.55	1.48 (0.78)	0.61 (0.41)	0.54 to 0.68	<0.001	0.856
Liver							
Reader A	0.74 ± 0.23	1.29 ± 0.41	1.10 (0.52)	0.54 (0.24)	0.49 to 0.59	<0.001	0.035
Reader B	0.75 ± 0.24	1.32 ± 0.41	1.20 (0.64)	0.56 (0.23)	0.53 to 0.60	<0.001	0.271
Vertebral body							
Reader A	1.73 ± 0.62	2.17 ± 0.72	2.05 (1.00)	0.40 (0.24)	0.36 to 0.43	<0.001	0.375
Reader B	1.78 ± 0.62	2.20 ± 0.72	2.16 ± 0.80	0.38 (0.24)	0.34 to 0.44	<0.001	0.692

Data are presented as mean ± standard deviation or median (interquartile range), as appropriate. Δpaired was calculated as LD-AiR minus LD-SAFIRE and is presented as median (interquartile range), reflecting within-patient paired differences in the LDCT group. The 95% CI refers to the nonparametric 95% confidence interval for the median paired difference. *p*1 denotes the paired comparison between LD-SAFIRE and LD-AiR, performed using the paired *t*-test or Wilcoxon signed-rank test, as appropriate. *p*2 denotes the between-group comparison between LD-AiR and SD-SAFIRE, performed using the independent-samples *t*-test or Mann–Whitney U test, as appropriate. All *p*-values were two-sided. SNR indicates signal-to-noise ratio.

**Table 5:** Comparison of CNR among LD-SAFIRE, LD-AiR, and SD-SAFIRE images.

CNR	LD-SAFIRE	LD-AiR	SD-SAFIRE	Δpaired	95% CI for median Δpaired	*p*1	*p*2
Lung							
Reader A	5.39 (7.42)	9.86 (15.15)	9.68 (11.30)	3.19 (6.85)	2.36 to 4.97	<0.001	0.625
Reader B	6.14 (8.41)	14.01 (19.14)	8.95 (16.60)	6.18 (10.36)	3.64 to 7.69	<0.001	0.389
Aorta							
Reader A	434.86 (495.52)	1085.52 (1660.04)	1052.48 (1778.72)	649.71 (1165.45)	363.79 to 858.23	<0.001	0.403
Reader B	485.26 (462.44)	1265.56 (1491.28)	1055.54 (1843.84)	804.10 (1104.65)	577.23 to 967.13	<0.001	0.515
Muscle							
Reader A	390.91 (421.79)	980.35 (1284.34)	1017.38 (1585.09)	606.61 (942.51)	467.68 to 786.68	<0.001	0.534
Reader B	393.69 (350.05)	895.94 (1192.36)	930.35 (1564.83)	524.44 (878.86)	421.25 to 757.80	<0.001	0.954
Liver							
Reader A	301.96 ± 148.62	692.30 (808.67)	588.68 (761.52)	470.53 (622.09)	247.54 to 592.79	<0.001	0.918
Reader B	301.83 (205.62)	889.05 (889.37)	628.67 (937.89)	565.86 (654.53)	397.24 to 650.70	<0.001	0.205
Vertebral body							
Reader A	186.15 (95.87)	258.19 (202.80)	314.31 (272.52)	85.17 (115.03)	65.07 to 114.89	<0.001	0.142
Reader B	194.42 (95.50)	326.48 ± 153.05	315.96 (222.06)	100.43 (99.23)	76.94 to 117.56	<0.001	0.485

Data are presented as mean ± standard deviation or median (interquartile range), as appropriate. Δpaired was calculated as LD-AiR minus LD-SAFIRE and is presented as median (interquartile range), reflecting within-patient paired differences in the LDCT group. The 95% CI refers to the nonparametric 95% confidence interval for the median paired difference. p1 denotes the paired comparison between LD-SAFIRE and LD-AiR, performed using the paired t-test or Wilcoxon signed-rank test, as appropriate. p2 denotes the between-group comparison between LD-AiR and SD-SAFIRE, performed using the independent-samples t-test or Mann–Whitney U test, as appropriate. All *p*-values were two-sided. CNR indicates contrast-to-noise ratio. CNR was calculated using a previously published noise-weighted formula applied in chest CT deep learning reconstruction studies. Because this formula differs from the conventional CNR definition, absolute CNR values should not be compared with conventional CNR values; only relative comparisons among reconstruction methods within this study should be interpreted.

### 3.6 Subjective image quality assessment

**Figure 3:**
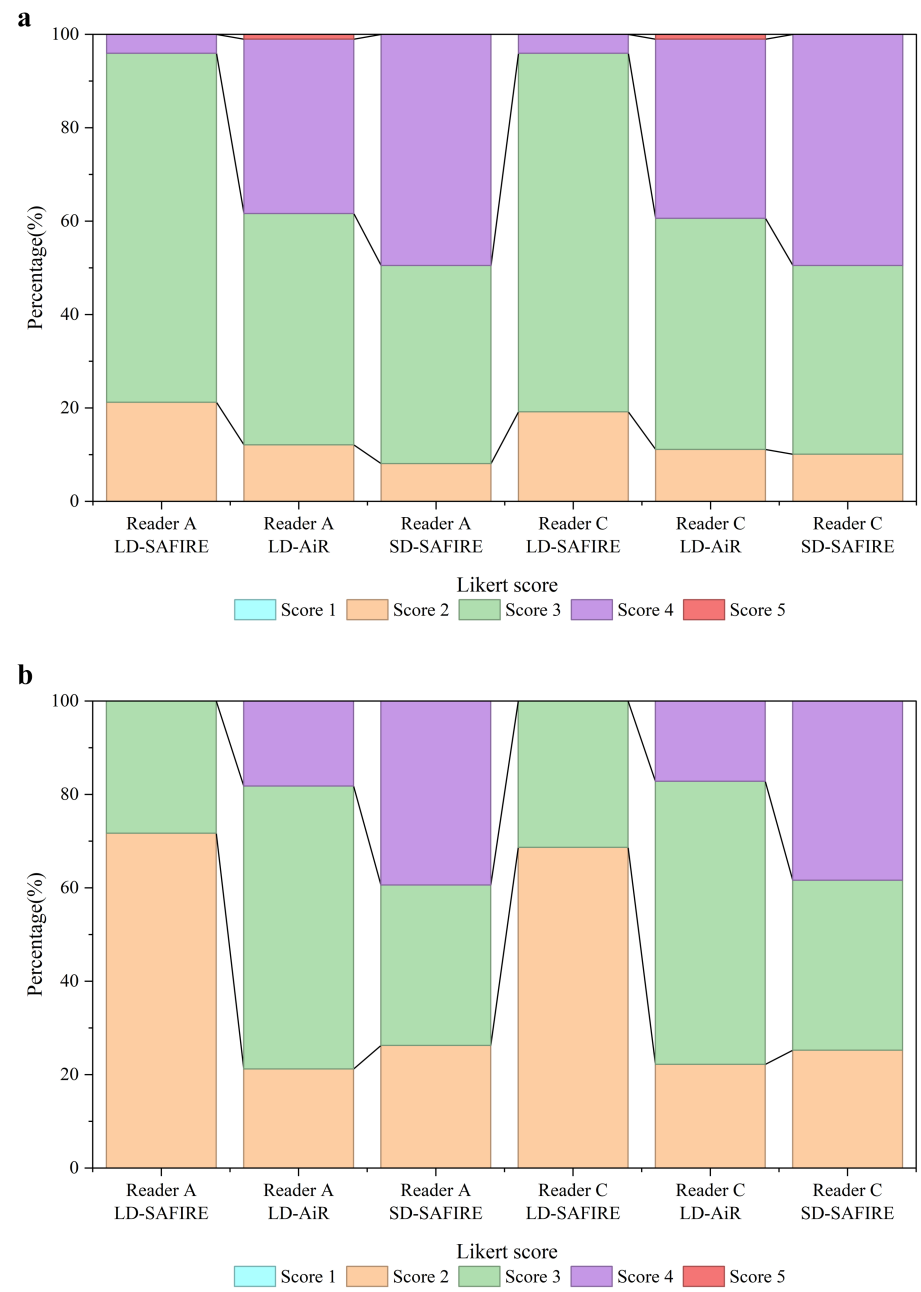
Stacked bar charts showing the distribution of subjective image-quality scores across reconstruction methods for lung parenchyma **(a)** and mediastinal soft tissue **(b)**. For both readers, AiR processing shifted the score distribution towards higher grades compared with LD-SAFIRE, thereby helping to explain the statistically significant differences despite similar median scores in some comparisons.

**Figure 4:**
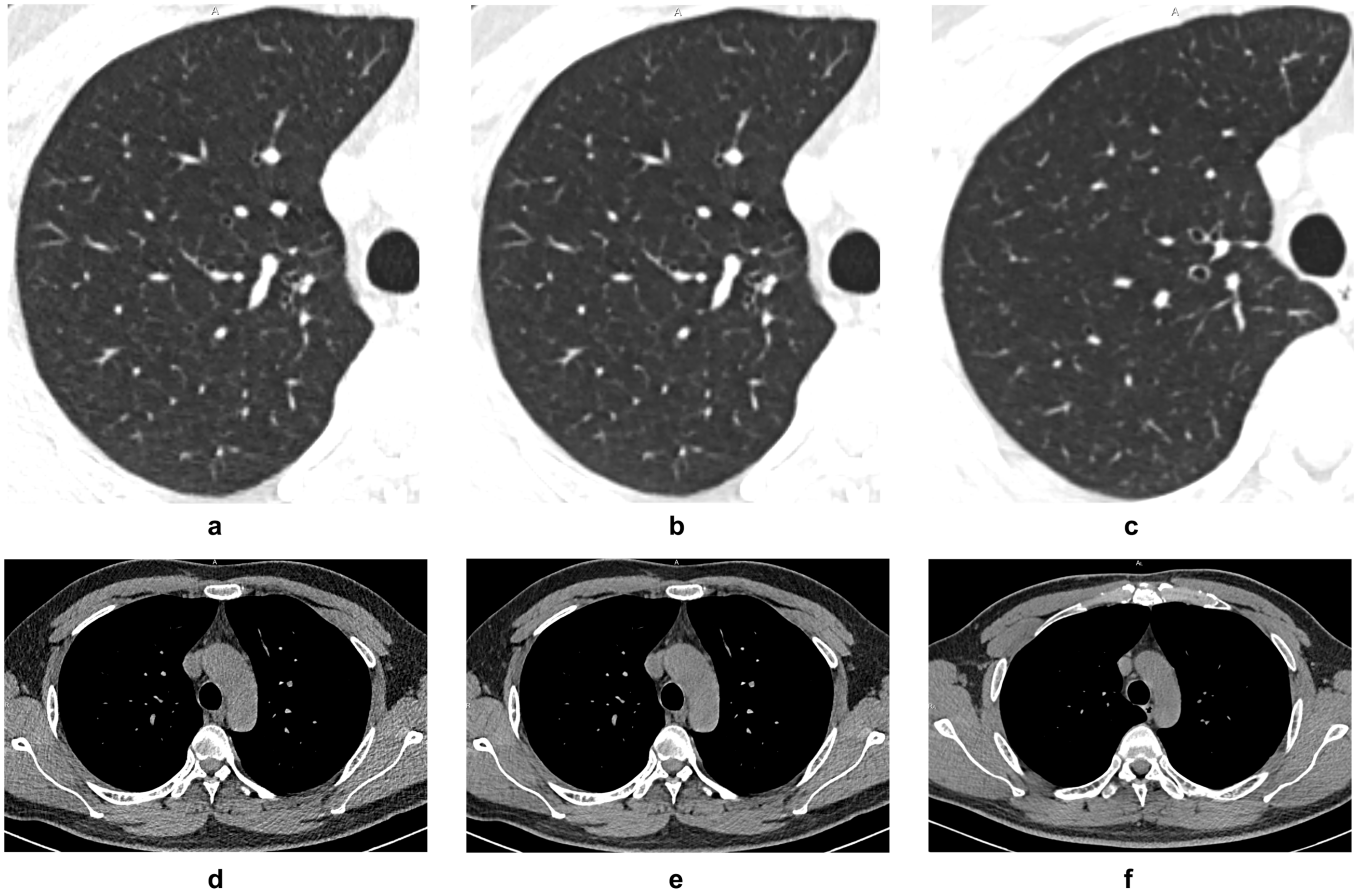
Representative axial unenhanced chest CT images from two participants with comparable body habitus. **(a–c)** Lung-window images: LD-SAFIRE **(a)**, LD-AiR **(b)**, and SD-SAFIRE **(c)**. **(d–f)** Mediastinal-window images: LD-SAFIRE **(d)**, LD-AiR **(e)**, and SD-SAFIRE **(f)**. Panels **a, b, d**, and **e** were from the same low-dose participant, whereas c and f were from another participant scanned with the standard-dose protocol. LD-AiR shows reduced image noise and improved delineation of pulmonary vessels and mediastinal soft-tissue interfaces compared with LD-SAFIRE, with an overall appearance similar to SD-SAFIRE.

Inter-reader agreement for the subjective assessment of image quality was almost perfect, with linearly weighted kappa (*κ*) values ranging from 0.899 to 0.971. Subjective quality scores for both lung parenchyma and mediastinal soft tissue were significantly higher for LD-AiR than for LD-SAFIRE (all *p* < 0.001). In addition, subjective scores for LD-AiR were largely comparable to those for SD-SAFIRE, with no significant differences in lung parenchymal or mediastinal soft-tissue image quality between the two groups (all *p* > 0.05). Although median scores were unchanged for lung parenchyma, the score distribution shifted toward higher ratings after AiR processing; for mediastinal soft tissue, both the median score and the score distribution shifted upward, with the median increasing from 2 to 3. Detailed results are presented in Table 6 and Figure 3, with complete score distributions shown in Supplementary Table S6. Representative images of lung and mediastinal windows are illustrated in Figure 4, where LD-AiR demonstrates a marked reduction in image noise and enhanced delineation of pulmonary and mediastinal structures when compared to LD-SAFIRE.

**Table 6:** Comparison of subjective image quality scores among LD-SAFIRE, LD-AiR, and SD-SAFIRE images.

Structure	LD-SAFIRE	LD-AiR	SD-SAFIRE	*p*1	*p*2
Lung parenchyma					
Weighted κ	0.899	0.971	0.939		
Reader A	3.0 (0.0)	3.0 (1.0)	3.0 (1.0)	<0.001	0.115
Reader C	3.0 (0.0)	3.0 (1.0)	3.0 (1.0)	<0.001	0.223
Mediastinal soft tissue					
Weighted κ	0.928	0.904	0.929		
Reader A	2.0 (1.0)	3.0 (0.0)	3.0 (2.0)	<0.001	0.098
Reader C	2.0 (1.0)	3.0 (0.0)	3.0 (1.0)	<0.001	0.065

Subjective image-quality scores are presented as median (interquartile range). Weighted κ indicates linearly weighted kappa for inter-reader agreement in ordinal subjective image-quality scores. *p*1 denotes the paired comparison between LD-SAFIRE and LD-AiR, performed using the Wilcoxon signed-rank test. *p*2 denotes the between-group comparison between LD-AiR and SD-SAFIRE, performed using the Mann–Whitney U test. All *p*-values were two-sided.

## 4 Discussion

In this retrospective study, we examined whether vendor-independent image-domain deep learning denoising, applied as post-processing, could enhance low-dose chest CT image quality on a CT scanner without native deep learning reconstruction capability. The main findings were as follows: First, compared to LD-SAFIRE, LD-AiR significantly reduced image noise and improved objective image quality metrics, including SD, SNR, and CNR, across multiple anatomical regions. Second, subjective image quality improved with LD-AiR compared to LD-SAFIRE. Third, the LDCT protocol was associated with an approximately 76% lower ED than the SDCT protocol. The SSDE analysis showed a consistent pattern: effective diameter was similar between groups, and SSDE remained significantly lower in the LDCT group. Most LD-AiR image-quality metrics did not differ significantly from those of SD-SAFIRE. However, because this comparison involved different patient groups and different acquisition protocols, it should be interpreted descriptively rather than as evidence of equivalence or non-inferiority. Together, these findings suggest that vendor-independent image-domain deep learning denoising is associated with improved image quality in routine low-dose chest CT on the tested CT platform and may be a practical option for CT systems lacking scanner-integrated deep learning reconstruction capability, although further diagnostic-performance and multicentre validation remains necessary.

In this study, on the evaluated CT system, vendor-independent image-domain deep learning denoising was associated with improved objective and subjective image quality in routine low-dose chest CT, with reduced image noise and increased SNR and CNR. The paired differences and corresponding 95% confidence intervals further indicated that the improvements were consistent in direction across evaluated anatomical regions, supporting the robustness of the observed image-quality changes beyond statistical significance alone. These findings align with previous studies showing that deep learning–based approaches can enhance the quality of low-dose chest CT images (8, 11, 12). Kim et al. (8) demonstrated that vendor-integrated deep learning reconstruction significantly reduced noise and improved image quality in low-dose chest CT performed at an average ED of 0.75 mSv on a high-performance CT platform, outperforming IR. More directly related to the present work, Nam et al. (11) reported in a clinical image-quality comparison study of 100 consecutive ultralow-dose noncontrast chest CT examinations that a vendor-agnostic post-processing algorithm yielded image quality metrics similar to, and in some respects better than, those of vendor-specific techniques. However, the present study was not designed to directly compare vendor-agnostic and vendor-specific reconstruction or post-processing techniques. In addition, Drews et al. (12) showed that AI-based post-processing significantly reduced noise and improved subjective image quality in non-contrast chest and abdominal CT, particularly for soft-tissue imaging. Unlike scanner-integrated deep learning reconstruction systems, AiR was applied as an image-domain post-processing algorithm to reconstructed DICOM images. This workflow may facilitate use on CT systems without native deep learning reconstruction capability, but the proprietary network architecture and training data were not available to the investigators; therefore, the present results should be interpreted as clinical image-quality observations rather than a mechanistic evaluation of the algorithm. From a broader medical imaging perspective, recent deep learning studies have emphasized that feature representation, attention mechanisms, data complexity, and model dependence may influence downstream image-analysis tasks, including structural MRI-based disease staging, multimodal MRI tumor classification and localization, brain MRI classification, and lung nodule segmentation (20–23). Although these studies involved different imaging tasks, they highlight the need to evaluate not only visual image quality and image-noise reduction, but also structural-detail preservation and downstream quantitative image interpretation after image-domain processing. In this context, the present study did not evaluate image texture, spatial-resolution preservation, radiomics features, quantitative imaging biomarkers, lesion detectability, or diagnostic performance, which should be addressed in future validation studies.

Recent clinical evidence further supports the feasibility of deep learning–based denoising strategies in CT imaging. Lanzafame et al. reported that deep learning–based denoising improved image quality metrics, including SNR and CNR, without negatively affecting diagnostic performance in cardiac CT imaging (13). However, unlike studies that incorporated diagnostic-performance assessment, the present study focused on image-quality endpoints and did not evaluate lesion detectability or diagnostic accuracy. Therefore, the observed improvements in image-quality metrics should not be interpreted as evidence that diagnostic performance, lesion detectability, or clinical decision-making is preserved. In addition, recent studies on deep learning reconstruction for low-dose chest CT have shown consistent improvements in image noise and overall image quality, supporting the broader applicability of deep learning–based techniques in thoracic imaging (24). Together, these findings suggest that vendor-independent image-domain post-processing may represent a practical approach for improving image quality in low-dose CT on the evaluated system; however, the present results reflect validation on a single platform and should not be interpreted as demonstrating cross-vendor generalizability.

Although several comparative studies in the literature evaluated scanner-integrated deep learning reconstruction embedded within the CT reconstruction pipeline, the present study investigated a vendor-independent image-domain post-processing algorithm applied on a single CT platform. These studies remain relevant as clinical benchmarks for the magnitude of achievable image quality improvement (8, 10). Nevertheless, direct comparisons should be interpreted with caution because image-domain denoising and scanner-integrated reconstruction operate at different stages of the image formation process and may differ in both information access and performance characteristics (5, 11, 12, 25).

In this study, LD-AiR and SD-SAFIRE showed no statistically significant differences in most attenuation, image-noise, SNR, and CNR measurements in this routine chest CT cohort, although a small number of reader-specific differences were observed. Meanwhile, the average ED was reduced from 3.29 mSv to 0.79 mSv, corresponding to an approximate 76% reduction. This dose difference should be interpreted as a protocol-level difference between the LDCT and SDCT acquisition protocols rather than as an isolated effect of image-domain denoising. These findings indicate that, on the tested platform, vendor-independent image-domain deep learning denoising was associated with image-quality metrics that were not significantly different from those observed with standard-dose IR for most assessed parameters at a substantially lower radiation dose. However, this observation should be interpreted cautiously. The comparison between LD-AiR and SD-SAFIRE was performed between different patient groups rather than within the same individuals. In addition, the LDCT and SDCT protocols differed in tube voltage, tube current–time product, pitch, and radiation output. Therefore, in the absence of formal equivalence or non-inferiority testing, non-significant findings should not be interpreted as evidence of equivalence. This interpretation aligns with the findings of Singh et al. (10), who reported that scanner-integrated deep learning reconstruction provided improved image noise and SNR compared to standard-dose IR while maintaining comparable detection rates for thoracic and abdominal lesions. Together, these findings suggest that vendor-independent image-domain post-processing may be a practical strategy for enhancing image quality in routine low-dose chest CT on the evaluated CT system, particularly for scanners without native deep learning reconstruction capability, although broader validation remains necessary (26).

Although statistically significant differences in attenuation values were identified between LD-SAFIRE and LD-AiR in several anatomical regions within the LDCT group, the supplementary analysis indicated that the magnitude of these differences was small for all tissue ROIs (Supplementary Table S4). Specifically, for both readers, the median ΔHU values ranged from −1.6 to −0.3 HU, with mean absolute differences between 0.8 and 1.7 HU observed in the lung, aorta, muscle, liver, and vertebral body. Notably, 98–100% of paired measurements remained within ±5 HU. These findings indicate that while attenuation shifts were statistically detectable, their magnitude was limited and likely not clinically meaningful for routine tissue ROI assessments. Previous studies have reported similar findings, in which deep learning-based reconstruction produced statistically significant but numerically small attenuation changes, generally within a few Hounsfield units (9). Such discrepancies may arise from the paired-comparison design, the considerable sample size, and the enhanced sensitivity of paired analyses in detecting minor numerical deviations. Furthermore, slight variability in ROI placement and subtle voxel redistribution due to image-domain post-processing may have also played a role in these findings. Therefore, the attenuation changes noted after LD-AiR processing should be considered quantitatively small and of limited clinical significance in tissue structures (27). Nevertheless, even small systematic attenuation or texture changes may be relevant for quantitative imaging applications or radiomics analysis, and the use of LD-AiR in such settings requires dedicated validation. Notably, background air demonstrated a relatively larger shift, indicating that the effect of post-processing on attenuation stability may not be uniform across different ROI types.

In this study, the magnitude of noise reduction achieved with LD-AiR was greater in soft-tissue structures, including the aorta, liver, and muscle, than in the lung and vertebral body, although measurable improvements were observed across all evaluated regions. This pattern is broadly consistent with findings reported by Jiang et al. (17). The greater benefit observed in soft-tissue structures may be related to the higher visibility of noise in relatively homogeneous soft-tissue regions, where noise suppression can more directly improve image uniformity and contrast. From a clinical perspective, these improvements may be particularly relevant for the visual assessment of mediastinal soft tissues on routine low-dose non-contrast chest CT (12, 28). Potential benefits for upper abdominal structures visible on routine chest CT are also plausible (12). Accordingly, on the tested platform, vendor-independent image-domain deep learning denoising may improve image quality for the assessment of mediastinal soft tissues and potentially visible upper abdominal structures, in addition to lung parenchyma (12), although broader validation is needed to determine whether these improvements translate into enhanced lesion detection or diagnostic performance.

The inter-reader agreement for thoracic diameter and scan-length measurements was excellent (ICC, 0.995–1.000), indicating that measurements based on clear anatomical landmarks were highly objective and reproducible. In contrast, inter-reader agreement for image noise measurements was more variable (ICC, 0.344–0.887). Compared to the higher agreement for noise measurements reported by Jiang et al. (17) (ICC, 0.716–0.937), this variability may be due to minor differences in ROI placement. Although the ROI locations were predefined, small spatial variations in ROI delineation between readers may have influenced the measured attenuation values and corresponding noise estimates. This effect may be more pronounced for pixel-wise SD-based noise measurements than for mean attenuation measurements, particularly in heterogeneous low-dose images and non-anatomical background air regions. Accordingly, measurements from regions with lower inter-reader agreement were interpreted cautiously, with emphasis placed on the consistency of paired changes across readers and image-quality metrics rather than on any single low-reliability measurement. For subjective image quality assessment, inter-reader agreement was almost perfect, with linearly weighted kappa (*κ*) values ranging from 0.899 to 0.971. This suggests that the visual image quality improvement associated with LD-AiR was consistently recognized by both readers, although these ordinal ratings should be interpreted together with the objective image-quality metrics. In addition, the 5-point Likert scale provided a practical ordinal assessment of perceived image quality but was inherently coarse and may not capture subtle differences in image texture or diagnostic confidence. Therefore, the subjective scores were interpreted as supportive visual assessments of perceived image quality rather than as quantitative or diagnostic endpoints.

This study has several limitations. First, this was a retrospective single-centre study performed on a single CT platform, and LD-AiR and SD-SAFIRE were compared between different patient groups and acquisition protocols; therefore, multicentre, multi-vendor, and prospective within-subject validation is needed. Second, the study focused on image-quality metrics in normal anatomical structures and did not assess lesion detectability, diagnostic confidence, diagnostic accuracy, or clinical decision-making. Third, some protocol-related factors could not be fully assessed retrospectively, including quantitative isocentering, vertical off-centering because only an anteroposterior localizer was obtained, individualized reconstruction field-of-view adjustment, and the use of a single SAFIRE strength/kernel/section-thickness setting, which may limit the generalizability of the findings to other reconstruction strengths, kernels, section thicknesses, or CT platforms. In addition, SSDE was estimated using the effective-diameter rather than attenuation-based method, and BMI data were incomplete. Finally, the proprietary nature of the AiR algorithm limited access to detailed network architecture and training-data information, which may affect technical reproducibility and external validation. Future prospective within-subject studies incorporating lesion-detectability assessment, diagnostic-performance evaluation, texture-preservation analysis, radiomics and quantitative imaging biomarkers, and multi-vendor validation are needed to further clarify the clinical role of image-domain denoising in low-dose chest CT.

## 5 Conclusion

Vendor-independent image-domain deep learning denoising improved image quality for low-dose chest CT on the evaluated CT platform, with significant image-noise reduction. In the descriptive between-group comparison, the LDCT protocol was associated with an approximately 76% lower radiation dose than the SDCT protocol, and most image-quality metrics of LD-AiR did not differ significantly from those of SD-SAFIRE. However, these findings should not be interpreted as evidence of equivalence or non-inferiority because of the retrospective between-group design, differences in acquisition protocols, and the absence of formal equivalence testing. These findings suggest the potential utility of this approach for image-quality improvement in low-dose chest CT on the evaluated system. Further prospective, multicentre studies incorporating lesion-detectability and diagnostic-performance assessment are needed to clarify its clinical applicability and generalisability across different CT platforms.

## Data Availability

The datasets analyzed in this study are not publicly available due to patient privacy and institutional/ethical restrictions. Requests to access the datasets should be directed to the corresponding author and will be considered subject to applicable institutional and ethical approvals.
