# Supplementary material for: Image-domain deep learning denoising for low-dose chest CT on a single 128-slice CT platform: a retrospective image-quality assessment
Source: Front Med (Lausanne). 2026 Jul 14;13:1888006. doi: 10.3389/fmed.2026.1888006 (PMC13407374; doi:10.3389/fmed.2026.1888006)
Supplement: Supplementary file 1 [file Data_Sheet_1.PDF]

Supplementary Material

Table S1. Comparison of thoracic diameters and scan length between the LDCT and SDCT groups

Parameter	LDCT group (n = 99)	SDCT group (n = 99)	Test statistic	*p*-value
Maximum transverse thoracic diameter, mm				
ICC	0.997	0.995		
Reader A	298.45 ± 25.27	296.16 ± 25.19	0.64	0.524
Reader B	298.51 ± 25.51	296.80 ± 25.22	0.47	0.637
Maximum anteroposterior thoracic diameter, mm				
ICC	0.997	0.999		
Reader A	230.40 (45.70)	219.50 (42.60)	-0.88	0.379
Reader B	230.50 (50.80)	219.30 (41.00)	-0.85	0.396
Scan length, mm				
ICC	1.000	0.999		
Reader A	311.90 ± 33.27	311.10 ± 29.20	0.18	0.858
Reader B	312.03 ± 33.35	311.29 ± 29.34	0.17	0.867

Note: Data are presented as mean ± standard deviation or median (interquartile range), as appropriate. ICC indicates inter-reader reliability. The test statistic denotes the t or Z value, as appropriate. All p-values were two-sided.

Table S2. Clinical indications for chest CT examinations in the LDCT and SDCT groups

Clinical indication	LDCT group (n = 99)	SDCT group (n = 99)	Total (n = 198)
Pulmonary nodule screening or follow-up	8 (8.1%)	11 (11.1%)	19 (9.6%)
Infection or inflammation assessment	59 (59.6%)	59 (59.6%)	118 (59.6%)
Chest symptom evaluation	5 (5.1%)	1 (1.0%)	6 (3.0%)
Tumor-related evaluation or follow-up	17 (17.2%)	18 (18.2%)	35 (17.7%)
Preoperative or routine inpatient assessment	3 (3.0%)	6 (6.1%)	9 (4.5%)
Other or unclear indications	7 (7.1%)	4 (4.0%)	11 (5.6%)
Total	99 (100.0%)	99 (100.0%)	198 (100.0%)

Note: Data are presented as n (%). Clinical indications were retrospectively extracted from the PACS/RIS examination request forms and categorized according to the predominant clinical reason for the examination. The distribution of clinical indications did not differ significantly between the LDCT and SDCT groups (Fisher–Freeman–Halton exact test, p = 0.444).

Table S3. Size-specific dose estimate (SSDE) based on effective diameter

Parameter	LDCT group (n = 99)	SDCT group (n = 99)	Test statistic	p-value
Effective diameter (cm)	26.19 (23.60–27.93)	25.36 (23.90–27.61)	U = 5220	0.429
CTDIvol (mGy)	1.43 (1.27–1.67)	6.01 (5.16–6.85)	U = 0	<0.001
SSDE (mGy)	1.98 (1.76–2.36)	8.78 (7.79–9.38)	U = 0	<0.001

Note: Data are presented as median (interquartile range). Effective diameter was calculated as the square root of the product of the mean anteroposterior and transverse diameters measured by two readers. SSDE was calculated by multiplying scanner-reported CTDIvol by the corresponding size-specific conversion factor for the 32-cm body CTDI phantom using the effective-diameter method described in AAPM Report 204. Between-group comparisons were performed using the Mann–Whitney U test. CTDIvol, volume CT dose index; LDCT, low-dose CT; SDCT, standard-dose CT; SSDE, size-specific dose estimate.

Table S4. Magnitude of paired attenuation differences between LD-SAFIRE and LD-AiR images by structure and reader

Structure	Reader A: Median ΔHU (Q1, Q3)	Reader A: Mean absolute difference, HU	Reader A: Within ±5 HU, n (%)	Reader B:Median ΔHU (Q1, Q3)	Reader B: Mean absolute difference, HU	Reader B: Within ±5 HU, n (%)
Lung	-0.3 (-0.7, 0.0)	0.8	98 (99.0%)	-0.3 (-0.8, 0.1)	0.8	98 (99.0%)
Aorta	-1.1 (-1.6, -0.7)	1.2	99 (100.0%)	-1.2 (-1.6, -0.8)	1.2	99 (100.0%)
Muscle	-1.0 (-1.7, -0.7)	1.2	99 (100.0%)	-1.3 (-1.8, -0.6)	1.3	99 (100.0%)
Liver	-1.6 (-2.3, -0.9)	1.7	99 (100.0%)	-1.4 (-2.2, -0.7)	1.6	99 (100.0%)
Vertebral body	-0.3 (-1.0, 0.5)	0.9	99 (100.0%)	-0.5 (-1.0, 0.3)	0.9	99 (100.0%)
Background air	5.0 (3.2, 6.8)	5.1	50 (50.5%)	5.2 (3.6, 6.8)	5.4	47 (47.5%)

Note. ΔHU was defined as LD-SAFIRE minus LD-AiR. This supplementary table is descriptive and is intended to show the magnitude of paired attenuation differences rather than to provide an alternative significance-testing framework. Hypothesis testing remains presented in Table S5. A difference within ±5 HU was considered small in magnitude for routine ROI attenuation comparison.

Table S5. Comparison of attenuation values among LD-SAFIRE, LD-AiR, and SD-SAFIRE images

CT attenuation, HU	LD-SAFIRE	LD-AiR	SD-SAFIRE	*p*1	*p*2
Lung					
ICC	0.847	0.849	0.813		
Reader A	-894.10 (34.10)	-892.70 (34.50)	-893.60 (35.90)	<0.001	0.853
Reader B	-892.90 (44.00)	-892.00 (43.40)	-895.30 (37.10)	<0.001	0.867
Aorta					
ICC	0.453	0.468	0.318		
Reader A	41.96 ± 10.14	43.14 ± 10.24	39.61 ± 9.71	<0.001	0.014
Reader B	42.38 ± 9.34	43.57 ± 9.39	41.28 ± 9.61	<0.001	0.091
Muscle					
ICC	0.419	0.437	0.773		

Reader A	55.40 (8.00)	56.40 (8.50)	51.80 (11.10)	<0.001	<0.001
Reader B	54.90 (11.10)	56.20 (10.90)	51.20 (12.40)	<0.001	<0.001
Liver					
ICC	0.788	0.802	0.753		
Reader A	54.30 (11.90)	55.40 (12.70)	53.70 (12.30)	<0.001	0.167
Reader B	53.30 (10.80)	55.30 (11.20)	54.10 (11.40)	<0.001	0.407
Vertebral body					
ICC	0.897	0.897	0.958		
Reader A	188.99 ± 56.41	198.70 (79.80)	171.76 ± 62.66	0.007	0.024
Reader B	193.95 ± 56.07	194.25 ± 56.17	176.64 ± 65.53	0.006	0.044
Background air					
ICC	0.515	0.510	0.516		
Reader A	-993.20 (35.10)	-1000.80 (32.90)	-997.40 (23.50)	<0.001	0.046
Reader B	-995.00 (23.20)	-1001.30 (22.60)	-996.70 (27.10)	<0.001	0.002

Note: Data are presented as mean ± standard deviation or median (interquartile range), as appropriate. ICC indicates inter-reader reliability. *p*1 denotes the paired comparison between LD-SAFIRE and LD-AiR, performed using the paired t-test or Wilcoxon signed-rank test, as appropriate. *p*2 denotes the between-group comparison between LD-AiR and SD-SAFIRE, performed using the independent-samples t-test or Mann–Whitney U test, as appropriate. All *p*-values were two-sided.

Table S6. Distribution of subjective scores across reconstruction methods by region and reader.

Region	Reader	Image set	Score 1 n (%)	Score 2 n (%)	Score 3 n (%)	Score 4 n (%)	Score 5 n (%)
Lung parenchyma	Reader A	LD-SAFIRE	0 (0.0%)	21 (21.2%)	74 (74.7%)	4 (4.0%)	0 (0.0%)
	Reader A	LD-AiR	0 (0.0%)	12 (12.1%)	49 (49.5%)	37 (37.4%)	1 (1.0%)
	Reader A	SD-SAFIRE	0 (0.0%)	8 (8.1%)	42 (42.4%)	49 (49.5%)	0 (0.0%)
	Reader C	LD-SAFIRE	0 (0.0%)	19 (19.2%)	76 (76.8%)	4 (4.0%)	0 (0.0%)
	Reader C	LD-AiR	0 (0.0%)	11 (11.1%)	49 (49.5%)	38 (38.4%)	1 (1.0%)
	Reader C	SD-SAFIRE	0 (0.0%)	10 (10.1%)	40 (40.4%)	49 (49.5%)	0 (0.0%)
Mediastinal soft tissue	Reader A	LD-SAFIRE	0 (0.0%)	71 (71.7%)	28 (28.3%)	0 (0.0%)	0 (0.0%)
	Reader A	LD-AiR	0 (0.0%)	21 (21.2%)	60 (60.6%)	18 (18.2%)	0 (0.0%)
	Reader A	SD-SAFIRE	0 (0.0%)	26 (26.3%)	34 (34.3%)	39 (39.4%)	0 (0.0%)
	Reader C	LD-SAFIRE	0 (0.0%)	68 (68.7%)	31 (31.3%)	0 (0.0%)	0 (0.0%)
	Reader C	LD-AiR	0 (0.0%)	22 (22.2%)	60 (60.6%)	17 (17.2%)	0 (0.0%)
	Reader C	SD-SAFIRE	0 (0.0%)	25 (25.3%)	36 (36.4%)	38 (38.4%)	0 (0.0%)

Note: Data are presented as n (%). Subjective scores were assigned independently by Readers A and C using a 5-point Likert scale for lung parenchyma and mediastinal soft tissue.

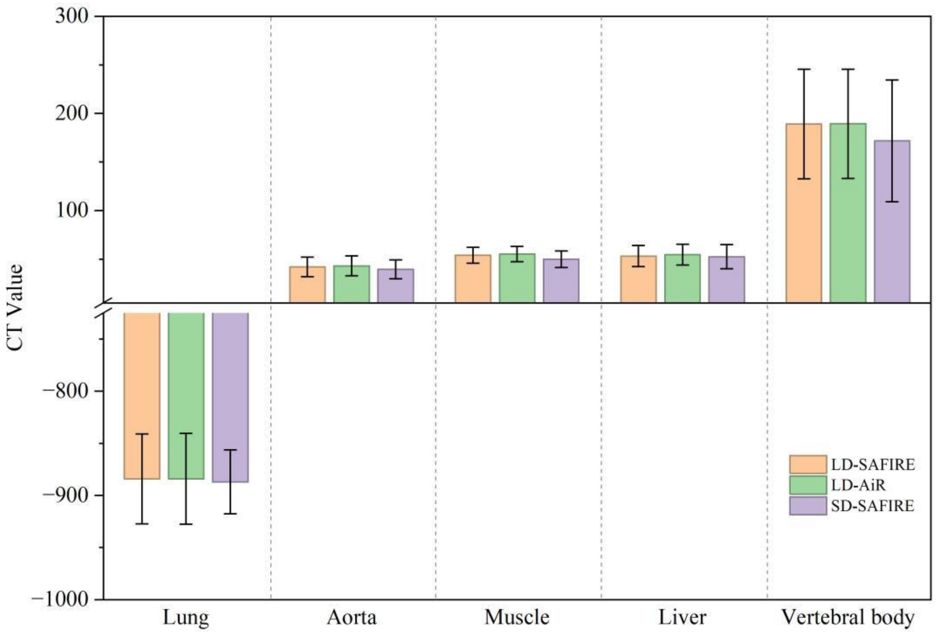

(a)

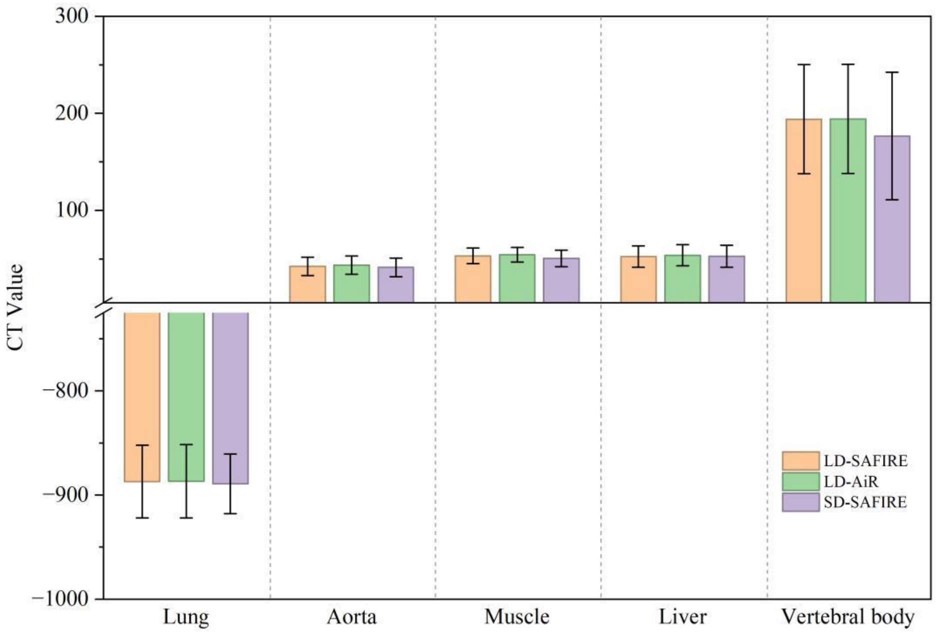

(b)

Figure S1. Attenuation measurements across reconstruction methods. Bar plots show attenuation values measured by Reader A (a) and Reader B (b) across anatomical regions in the LD-SAFIRE, LD-AiR, and SD-SAFIRE image sets. Error bars indicate standard deviation.

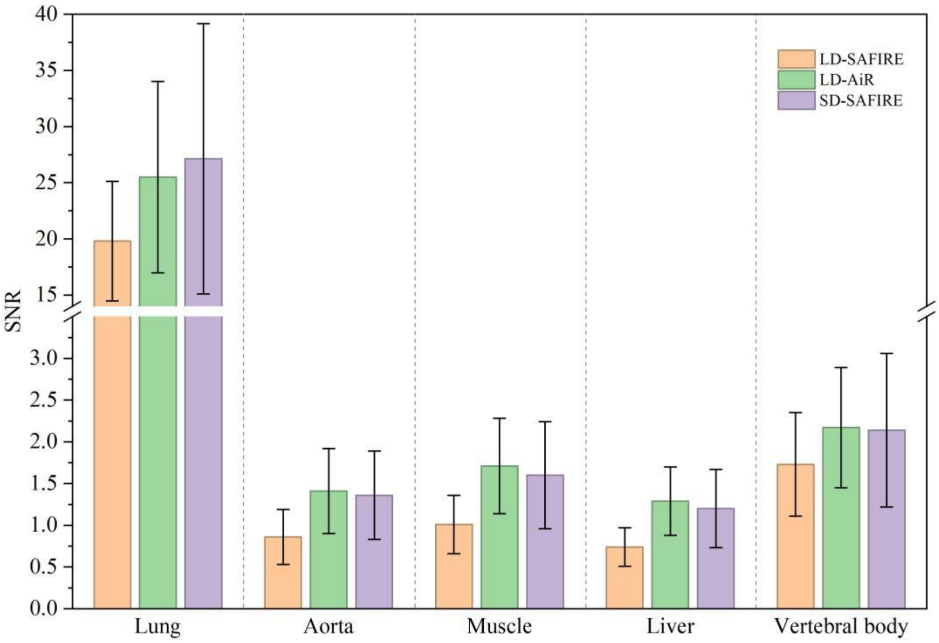

(a)

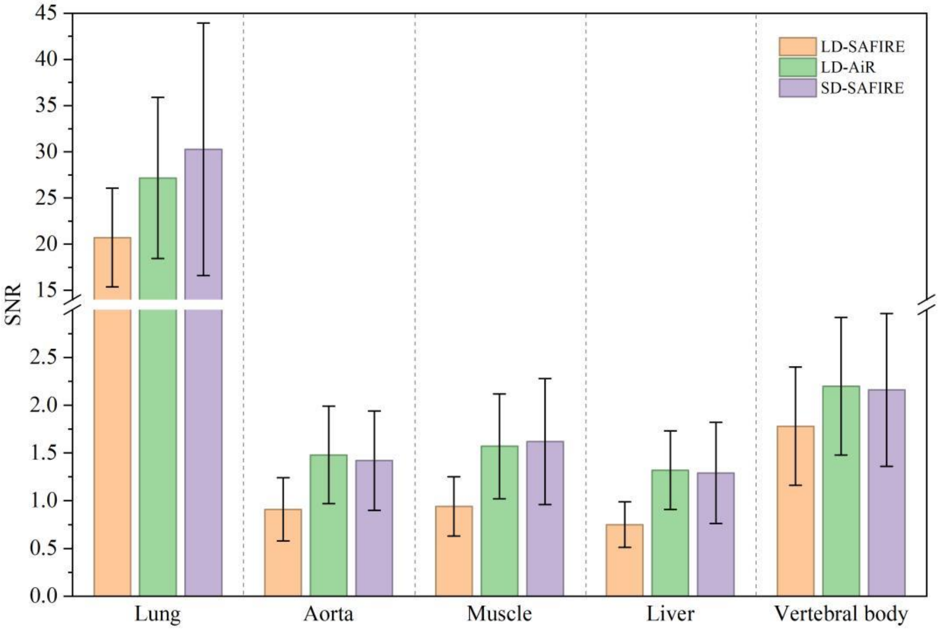

(b)

Figure S2. SNR across reconstruction methods. Bar plots show signal-to-noise ratio measured by Reader A (a) and Reader B (b) across anatomical regions in the LD-SAFIRE, LD-AiR, and SD-SAFIRE image sets. Error bars indicate standard deviation.

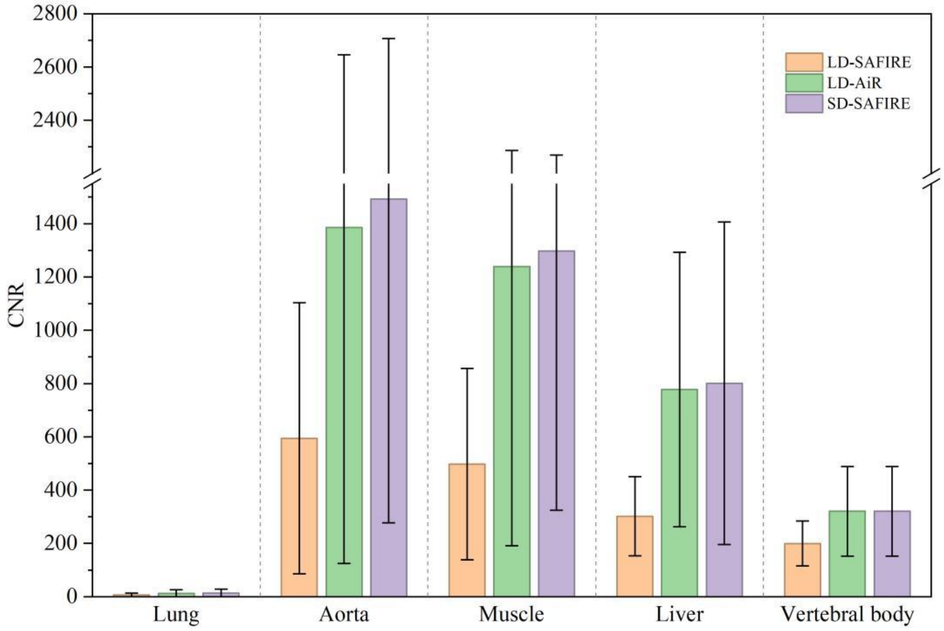

(a)

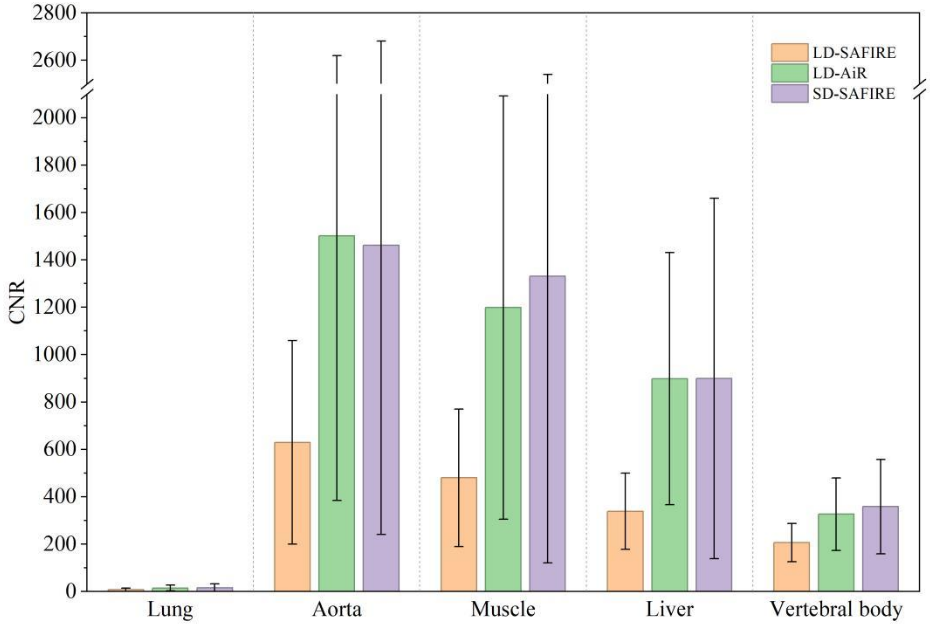

Figure S3. CNR across reconstruction methods. Bar plots show contrast-to-noise ratio measured by Reader A (a) and Reader B (b) across anatomical regions in the LD-SAFIRE, LD-AiR, and SD-SAFIRE image sets. Error bars indicate standard deviation. CNR was calculated using a previously published noise-weighted formula and should be interpreted only for relative comparisons within this study.
